# The complete chloroplast genome of *Sibbaldia aphanopetala* (Rosaceae: Potentilleae)

**DOI:** 10.1080/23802359.2020.1756945

**Published:** 2020-05-12

**Authors:** Xiao-Hui Zhang, Khasbagan, Qin-Qin Li

**Affiliations:** College of Life Science and Technology, Inner Mongolia Normal University, Hohhot, Inner Mongolia, China

**Keywords:** Chloroplast genome, Potentilleae, Rosaceae, *Sibbaldia aphanopetala*

## Abstract

The complete chloroplast genome of *Sibbaldia aphanopetala* reported herein was a circular DNA molecule of 153,491 bp in length. The genome had a typical quadripartite structure, consisting of a pair of inverted repeats (IRa and IRb: 25,611 bp) separated by a large single-copy region (LSC: 84,244 bp) and a small single-copy region (SSC: 18,025 bp). The cp genome encoded a set of 129 genes, containing 84 protein-coding genes, 37 tRNA genes, and eight rRNA genes. Phylogenetic analysis indicated that *S. aphanopetala* was sister to *S. procumbens*.

*Sibbaldia aphanopetala* Hand.-Mazz. [synonym: *S. procumbens* L. var. *aphanopetala* (Hand.-Mazz.) T. T. Yü et C. L. Li] belongs to the family Rosaceae Juss., subfamily Rosoideae (Juss.) Arn., tribe Potentilleae Sweet. It is a perennial herb distributed in the following provinces of China: Gansu, Qinghai, Shaanxi, Sichuan, Xizang, and Yunnan (Li et al. 2003). It is used as traditional herbal medicine to treat cough and menstrual disorders, to control bruising and to reduce swelling (Yü and Li 1985).

Fresh leaves of *S. aphanopetala* were collected from Kangding, Sichuan province, China. Voucher specimen (no. Li QQ YB720) was deposited in the herbarium of Inner Mongolia Normal University (NMTC). Total genomic DNA was isolated using the CTAB protocol of Doyle and Doyle (1987). The library with insert size of 300 bp fragments was constructed and sequenced using the Illumina HiSeq platform in Novogene (Nanjing, China). Illumina paired-end sequencing generated a total of 37,022,002-bp raw reads after removing adapters. The raw reads were then used to assemble the cp genome using NOVOPlasty (Dierckxsens et al. 2017), with ribulose-1, 5-bisphosphate carboxylase/oxygenase (*rbcL*) gene from *S. procumbens* (GenBank accession no. KY419935) as the seed. Chloroplast genome annotation was performed using transferring annotations in Geneious Prime (Kearse et al. 2012), with the cp genome of *Farinopsis salesoviana* (Steph.) Chrtek et Soják (GenBank accession no. MT017928) as the reference. Where necessary, the positions of start and stop codons and boundaries between introns and exons were manually corrected. The annotated complete cp genome of *S. aphanopetala* was deposited in GenBank under the accession no. MT178810. The complete cp genome of *S. aphanopetala* was a circular DNA molecule of 153,491 bp in length. The genome had a typical quadripartite structure, consisting of a pair of inverted repeats (IRa and IRb: 25,611 bp) separated by a large single-copy region (LSC: 84,244 bp) and a small single-copy region (SSC: 18,025 bp). The overall GC content was 37.2%. The IR regions had a higher GC content (42.7%) than the LSC (35.1%) and SSC (31.1%) regions. The cp genome encoded a set of 129 genes, containing 84 protein-coding genes, 37 tRNA genes, and eight rRNA genes.

To investigate the phylogenetic position of *S. aphanopetala*, the cp genome sequences of 32 Potentilleae species plus five *Rosa* species were aligned with MAFFT version 7.450 (Katoh and Standley 2013) and then trimmed properly using trimAL version 1.4 (Capella-Gutiérrez et al. 2009). A maximum likelihood (ML) tree was inferred using RAXML version 8 (Stamatakis 2014), with the combined rapid bootstrap (1000 replicates) and search for ML tree (the ‘-f a’ option). The GTRGAMMA model was used in the ML analysis. Phylogenetic tree indicated that *S. aphanopetala* was sister to *S. procumbens* (Figure 1).

**Figure 1.**
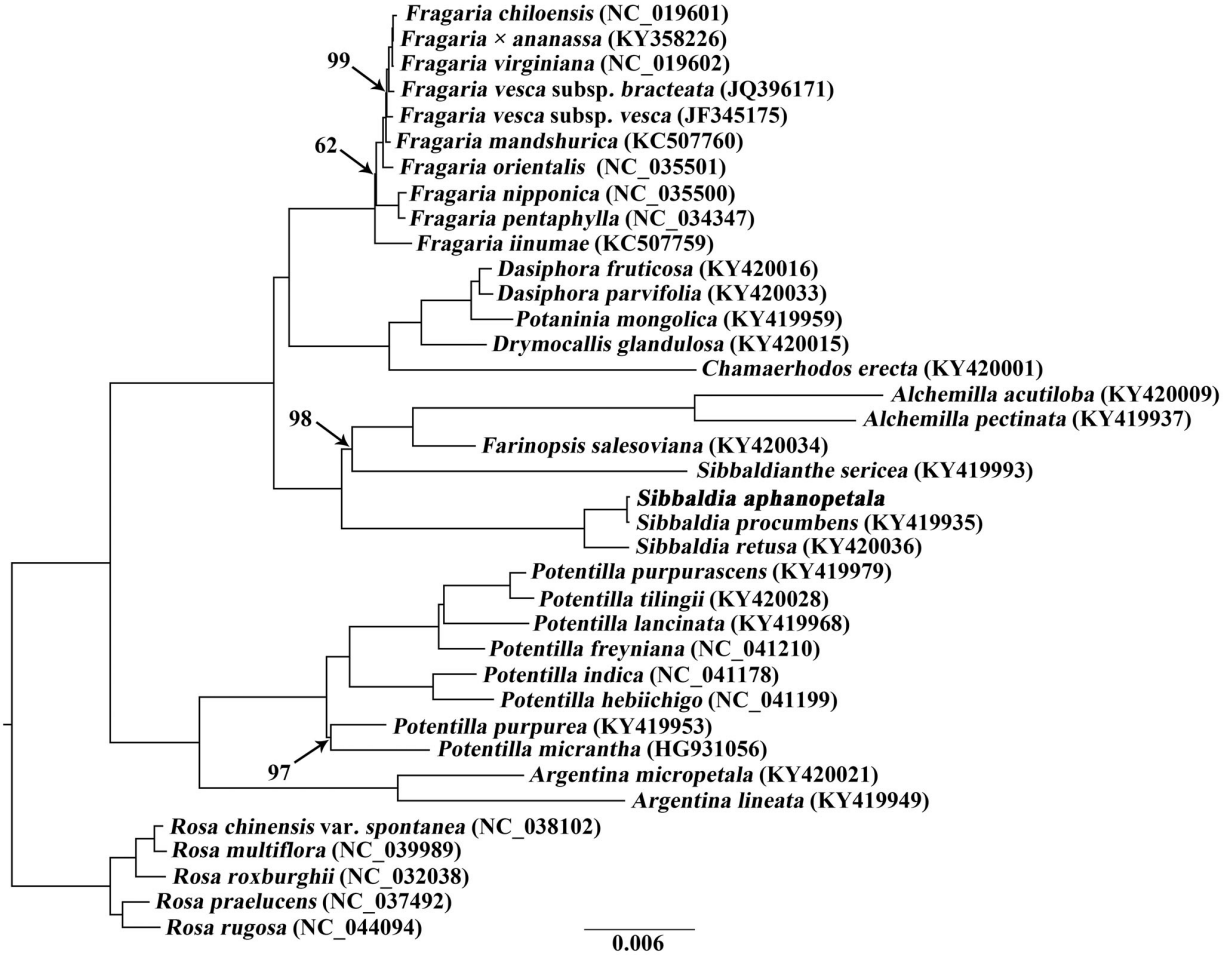
Maximum likelihood (ML) tree based on the cp genome sequences from 32 Potentilleae taxa plus five Rosa species as outgroups. Values along branches correspond to ML bootstrap percentages (only values <100% are shown).

## Data Availability

The authors confirm that the data supporting the finding of this study are available within its supplementary material.
